# Coffee Agroforests Remain Beneficial for Neotropical Bird Community Conservation across Seasons

**DOI:** 10.1371/journal.pone.0065101

**Published:** 2013-09-18

**Authors:** Sonia M. Hernandez, Brady J. Mattsson, Valerie E. Peters, Robert J. Cooper, C. Ron Carroll

**Affiliations:** 1 Odum School of Ecology, University of Georgia, Athens, Georgia, United States of America; 2 Daniel B. Warnell School of Forestry and Natural Resources, University of Georgia, Athens, Georgia, United States of America; University of Toronto, Canada

## Abstract

Coffee agroforestry systems and secondary forests have been shown to support similar bird communities but comparing these habitat types are challenged by potential biases due to differences in detectability between habitats. Furthermore, seasonal dynamics may influence bird communities differently in different habitat types and therefore seasonal effects should be considered in comparisons. To address these issues, we incorporated seasonal effects and factors potentially affecting bird detectability into models to compare avian community composition and dynamics between coffee agroforests and secondary forest fragments. In particular, we modeled community composition and community dynamics of bird functional groups based on habitat type (coffee agroforest vs. secondary forest) and season while accounting for variation in capture probability (i.e. detectability). The models we used estimated capture probability to be similar between habitat types for each dietary guild, but omnivores had a lower capture probability than frugivores and insectivores. Although apparent species richness was higher in coffee agroforest than secondary forest, model results indicated that omnivores and insectivores were more common in secondary forest when accounting for heterogeneity in capture probability. Our results largely support the notion that shade-coffee can serve as a surrogate habitat for secondary forest with respect to avian communities. Small coffee agroforests embedded within the typical tropical countryside matrix of secondary forest patches and small-scale agriculture, therefore, may host avian communities that resemble those of surrounding secondary forest, and may serve as viable corridors linking patches of forest within these landscapes. This information is an important step toward effective landscape-scale conservation in Neotropical agricultural landscapes.

## Introduction

Habitat loss and fragmentation continue to be the leading threats to biodiversity in the Neotropics [1]. Previous conservation efforts have focused on protecting large tracts of undisturbed forest, a solution that is often not possible or feasible. In response, the term “gardenification” was coined to emphasize a philosophical shift in conservation away from total reliance upon unpopulated preserves and towards encouraging people to appropriately manage their “gardens”, or small parcels of privately owned land, to improve biodiversity [2]. In contrast with more traditional conservation approaches that emphasize protection of uninhabited landscapes, conserving ecological integrity within human-dominated landscapes can be achieved through focusing on biodiversity benefits to humanity, such as an ‘ecosystem services’ approach or the use of innovative markets for specialized products, e.g. shade-grown coffee [3]. As an extension of gardenification, sustainable agriculture has been forwarded as a means to ensure that private land, while providing economic support for people, sustains biodiversity and buffers protected areas [4]. For example, there is evidence that coffee plantations managed under a floristically and structurally diverse tree canopy provide important habitat for diverse avian communities [5], [6], [7]. Thus, shade-grown coffee may be viewed as a surrogate for forest habitats in the tropics by maintaining avian communities that reflect those found in large tracts of forest (henceforth, habitat-surrogate hypothesis).

However, some authors have cautioned that the conservation value of shade-coffee may be overstated and that although avian biodiversity may be conserved in these systems, bird assemblages are not identical to those in secondary forests [8], [9]. Furthermore, most coffee studies have not sufficiently quantified observer bias or detectability differences between habitats, making abundance estimates problematic [8], [10]. Many of the pioneering studies assessing differences between coffee agroforestry systems and secondary forests compared the two habitat types using indices of species diversity (e.g. Shannon diversity index) but did not present habitat-specific community compositions or consider factors affecting detectability or capture probability [11], [12]. Furthermore, as tropical avian community dynamics are highly dependent on insect and plant phenology, studies comparing shade-coffee and secondary forests should span multiple seasons. Thus, a more informative approach to determine the suitability of shade-coffee for protecting forest bird species would be to describe and compare the dynamics of avian communities between shade-coffee and forest with respect to the changing environmental conditions across seasons [13], [14] while incorporating the factors influencing detectability of species such as breeding phenology, foraging preferences and habitat vegetation structure [15], [16].

The objective of our study was to estimate and compare avian community composition and dynamics between coffee agroforests and secondary forests while accounting for seasonal dynamics and differences in detectability between habitats and among species functional groups. We chose to model avian community composition with respect to functional groups (e.g. dietary guilds) to reflect the ecosystem services provided by birds in agroforestry systems including pest control by insectivores and seed dispersal by frugivores, and such an ecosystem-services approach has been promoted for biodiversity conservation in human-dominated landscapes [3].

We predicted that turnover in the bird species community would differ by habitat type, because shade-coffee plantations can have a higher temporal patchiness in food resource availability compared to forests, and birds are highly mobile species that track resources [17], [18]. For example, some food resources in coffee plantations are dependent upon the annual crop cycle and relate to tilling, pruning of trees or farmer selection in planting of crop/shade tree species that affect insect and fruit phenology. Neotropical secondary forests, in comparison, have a higher plant species richness and therefore higher spatio-temporal predictability of food resource availability [19]. Based on these habitat type comparisons, we predict that managed shade-coffee plantations, when compared to secondary forest, provide a more dynamic source of food resources for birds across seasons, leading to lower patch persistence by birds between seasons.

## Materials and Methods

### Study Area

Our study took place in the San Luis valley, located in the northwest region of Costa Rica approximately 7 km from the town of Santa Elena in the Monteverde region, in the province of Puntarenas. In the Monteverde region life zones change dramatically with only small changes in altitude, such that six life zones occur within a 600 m elevational range [20]. In addition to altitude, topographic position and exposure to trade winds and trade-wind driven clouds and rain shape a variety of microclimates in the Monteverde region [21]. Mean annual precipitation in the Monteverde region is estimated at 2500 mm; however, precipitation measurements underestimate moisture from wind-driven clouds. Two distinct weather seasons are defined in the region: (1) *wet season*- May to November; mornings are clear, yet cloud accumulation throughout the day results in rain in the afternoon and evening, and (2) *dry season*- December to April; begins with very strong trade winds, clouds and wind-driven rain giving way to moderate winds, clear skies and wind-driven mist, particularly at night and early morning [21]. The San Luis valley is located on the Pacific, leeward side of the volcanic Cordillera de Tilarán mountain range, and therefore the valley experiences a more well-defined, i.e. longer and drier, dry season than the broader Monteverde region [21]. Despite the long dry season, due to its high elevation, the forests of the San Luis valley are primarily evergreen (<10% of the canopy is leafless during the dry season), and have a moderate epiphyte diversity and abundance. The understory in these forests is fairly open with few shrubs and tree saplings [20].

**Figure 1:**
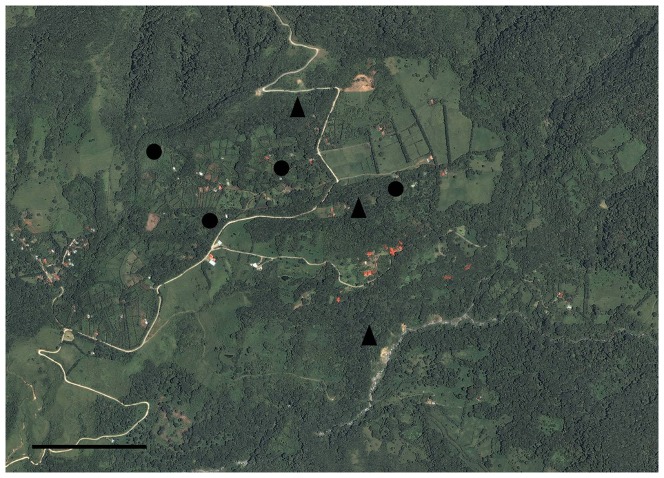
Study area as viewed from an airplane in the San Luis Valley near the Monteverde Reserve in Costa Rica. Shade coffee sites are indicated by circles, and secondary forest sites are indicated by triangles. Black bar indicates approximately 1 km.

Seven sites in the upper San Luis valley (10°16′N, 84°47′W; 950–1100 m elevation) were selected to represent the two habitat types (shade-coffee and secondary forest) (Fig. 1). Four study sites were privately owned, shade-grown coffee plantations of approximately 1–2 ha, and the other three sites were located within large patches (>5 ha) of secondary forest. All forest patches used in the study were approximately 50–75 yrs old. All coffee sites share a similar history, with coffee mainly being sun grown in the region during the 60's and 70's, but with the promotion of conservation-friendly agriculture in the region, most coffee in the San Luis valley now incorporates many shade trees. All coffee sites shared the following management practices: a moderate canopy cover [percent canopy cover range: 31–60%; mean tree height: 7.9 m (range: 6.9–9.6 m); and mean distance between trees: 7.3 m (range: 6.7–8.3 m)], a high species richness of shade trees (mean: 21 species ha^−1^), no pesticide use, and mechanical removal of weeds via machete. All secondary forest sites also shared similar vegetation structural characteristics, with percent canopy cover ranging from 87–91%, a mean tree height of 24.2 m (range: 16.6–31.0 m), a mean distance between trees of 2.6 m (range: 1.3–4 m), and a mean of 59 tree species ha^−1^. All study sites were separated by at least 250 m (max distance: 1.5 km), and all shade coffee sites were located within an agricultural matrix typical of those found throughout the neotropics, that included small forest patches, monoculture windbreaks (*Croton niveus* Jacq. or *Montanoa guatemalensis* B. L. Rob), and cattle pasture.

### Avian capture events and seasons

Birds were sampled in study sites across four pre-defined seasons from 2005–2008. Pre-defined seasons represented combinations of two biologically relevant factors: climatic cycle (i.e. alternating wet and dry seasons) and reproductive activity (peak breeding and non-peak breeding seasons; Table 1). The annual cycle of seasons was defined in the following chronological sequence: (1) dry, non-peak breeding (December–January); (2) dry, peak breeding (February–April); (3) wet, peak breeding (May–August) and (4) wet, non-peak breeding (September–November). These seasons distinguish the biologically relevant time periods for birds in this region [22]. For modeling purposes migratory birds were assumed to be absent during season 3 (i.e. wet, peak breeding).

**Table 1:** Annual avian capture bouts during four seasons in seven sites near San Luis, Costa Rica, 2005–2008.

	Dry						Wet					
	Non-peak breeding			Peak breeding			Peak breeding			Non-peak breeding		
Sites	2005	2006–7	2007–8	2005	2006	2007	2005	2006	2007	2005	2006	2007
Shade coffee												
Gilberth	**×**			**×**		**×**	**×**	**×**	**×**	**×**		
Joel	**×**	**×**		**×**			**×**	**×**		**×**		
Alvaro	**×**	**×**		**×**		**×**	**×**	**×**		**×**		
Vargas				**×**			**×**					
Secondary forest												
Zapote	×		×	×		×	×	×	×	×		
Nenes	×		×	×		×	×	×		×		
Pena	×		×	×		×	×	×	×	×		

The annual cycle of seasons follow a chronological sequence: (1) dry, non-peak breeding (December–January); (2) dry, peak breeding (February–April); (3) wet, peak breeding (May–August); and (4) wet, non-peak breeding (September–November). An×in a cell indicates that a particular site was sampled during a particular season-year combination.

Within each season, birds were sampled across multiple days in a site using mist-nets, and following a standard methodology [23]. Eight to fourteen nylon mesh mist-nets (38-mm mesh, 2 m high×either 6 or 9 m in length) were placed within a 1-ha area of each site, and at least 20 m from any bordering habitat type to ensure that captured birds were representative of the habitat type in which they were captured. The number of nets placed per site was multiplied by each net's length to ensure that total net length per site was equal. During each capture day, nets were opened starting between 0530 and 0700 h, and nets remained open until 1300 to 1400 h, depending on weather conditions (i.e. mist-nets were closed during periods of heavy rain, or when wind speed exceeded 20 kph). During each season, mist-nets were placed in two sites at a time for 4- to 7-d periods, and capture days were alternated between the two sites on consecutive days to prevent net shyness (i.e. decrease in daily capture probability) [24], [25]. Efforts were made to sample all seven sites during each sampling period, although this was not always feasible. Captured birds were extracted from mist-nets at 30-min intervals, and birds were processed immediately to minimize holding time before they were released near the point of capture. All birds >10 g were held for biological sample collection as part of a concurrent study [26] and were processed within 20 min of extraction from the nets. Birds were identified to species according to the available field guides [27], [28]. Following published species accounts, all bird species captured were classified according to the following life history attributes: migratory behavior (migratory, non-migratory), foraging strata preference (ground/understory or middle/upper canopy), and diet (frugivore, omnivore, insectivore; Table 2) [27], [28]. All avian capture and handling techniques were reviewed and approved by the University of Georgia's Animal Care and Use Committee (A2008 03-061-Y3-A0).

**Table 2:** Total number birds captured in shade coffee and secondary forest sites near San Luis, Costa Rica, 2005–2008.

Common name	Scientific name	Migratory status	Diet	Foraging	Forest	Coffee
Alder Flycatcher	*Empidonax alnorum*	M	O	Y	0	3
Baltimore Oriole	*Icterus galbula*	M	O	Y	0	4
Barred Antshrike	*Thamnophilus doliatus*	R	I	Y	0	2
Northern Barred-Woodcreeper	*Dendrocolaptes sanctithomae*	R	I	Y	6	0
Black-and-white Warbler	*Mniotilta varia*	M	I	Y	1	3
Black-headed Nightingale-Thrush	*Catharus mexicanus*	R	O	Y	1	0
Blue-crowned Motmot	*Momotus momota*	R	O	N	16	26
Blue-gray Tanager	*Thraupis episcopus*	R	O	N	0	5
Boat-billed Flycatcher	*Megarynchus pitangua*	R	O	N	0	1
Brown Jay	*Psilorhinus morio*	R	O	N	0	1
Buff-throated Saltator	*Saltator maximus*	R	O	Y	1	11
Canada Warbler	*Cardellina canadensis*	M	I	N	1	2
Chiriqui Quail-Dove	*Geotrygon chiriquensis*	R	O	Y	0	1
Clay-colored Thrush	*Turdus grayi*	R	O	Y	23	84
Common Bush-Tanager	*Chlorospingus ophthalmicus*	R	O	Y	10	0
Dusky-capped Flycatcher	*Myiarchus tuberculifer*	R	O	Y	4	22
Emerald Toucanet	*Aulacorhyncus prasinus*	R	O	N	5	9
Eye-ringed Flatbill	*Rhynchocyclus brevirostris*	R	O	N	1	0
Golden-crowned Warbler	*Basileuterus culicivorus*	R	O	Y	25	1
Grayish Saltator	*Saltator coerulescens*	R	O	Y	0	2
Great Kiskadee	*Pitangus sulphuratus*	R	O	N	0	1
House Wren	*Troglodytes aedon*	R	I	Y	4	19
Keel-billed Toucan	*Ramphastos sulfuratus*	R	O	N	1	0
Kentucky Warbler	*Geothlypis formosa*	M	I	Y	10	1
Least Flycatcher	*Empidonax minimus*	M	O	Y	0	1
Lesser Elaenia	*Elaenia chiriquensis*	R	O	N	0	1
Lesser Greenlet	*Hylophilus decurtatus*	R	O	N	9	4
Long-tailed Manakin	*Chiroxiphia linearis*	R	F	N	151	49
Louisiana Waterthrush	*Parkesia motacilla*	M	I	N	0	1
Paltry Tyrannulet	*Zimmerius vilissimus*	R	O	N	0	4
Mountain Elaenia	*Elaenia frantzii*	R	O	N	0	1
Mountain Thrush	*Turdus plebejus*	R	O	Y	0	5
Ochre-bellied Flycatcher	*Mionectes oleagineus*	R	O	Y	33	11
Olivaceous Woodcreeper	*Sittasomus griseicapillus*	R	I	Y	10	0
Olive-striped Flycatcher	*Mionectes olivaceus*	R	O	Y	3	2
Orange-bellied Trogon	*Trogon aurantiiventris*	R	O	N	1	0
Orange-billed Nightingale-Thrush	*Catharus aurantiirostris*	R	O	Y	90	73
Ovenbird	*Seiurus aurocapilla*	M	I	Y	10	5
Philadelphia Vireo	*Vireo philadelphicus*	M	O	N	0	2
Plain Wren	*Thryothorus modestus*	R	I	Y	10	29
Plain Xenops	*Xenops minutus*	R	I	N	1	0
Red-crowned Ant-Tanager	*Habia rubica*	R	O	Y	19	0
Red-eyed Vireo	*Vireo olivaceus*	M	O	Y	0	2
Rufous-breasted Wren	*Thryothorus rutilus*	R	I	Y	1	0
Ruddy Woodcreeper	*Dendrocincla homochroa*	R	I	N	48	6
Rufous-browed Peppershrike	*Cyclarhis gujanensis*	R	I	N	0	3
Rufous-capped Warbler	*Basileuterus rufifrons*	R	O	Y	55	41
Rufous-and-white Wren	*Thryothorus rufalbus*	R	I	Y	62	49
Passerini's Tanager	*Ramphocelus passerinii*	R	O	Y	1	0
Silver-throated Tanager	*Tangara icterocephala*	R	O	N	0	1
Streak-headed Woodcreeper	*Lepidocolaptes souleyetii*	R	I	N	2	5
Swainson's Thrush	*Catharus ustulatus*	M	O	Y	13	24
Tennessee Warbler	*Oreothlypis peregrina*	M	O	N	1	3
Wedge-billed Woodcreeper	*Glyphorynchus spirurus*	R	I	Y	0	1
Eastern Wood-Pewee	*Contopus virens*	M	I	Y	0	1
White-eared Ground-Sparrow	*Melozone leucotis*	R	O	Y	80	79
White-throated Thrush	*Turdus assimilis*	R	O	Y	27	11
White-tipped Dove	*Leptotila verreauxi*	R	O	Y	5	8
Wilson's Warbler	*Cardellina pusilla*	M	I	N	4	6
Wood Thrush	*Hylocichla mustelina*	M	O	Y	11	10
Worm-eating Warbler	*Helmitheros vermivorum*	M	I	Y	2	0
Yellow-bellied Flycatcher	*Empidonax flaviventris*	M	I	N	0	4
Yellow Tyrannulet	*Capsiempis flaveola*	R	O	Y	2	1
Yellow-bellied Elaenia	*Elaenia flavogaster*	R	O	Y	0	2
Yellow-billed Cacique	*Amblycercus holosericeus*	R	O	Y	1	4
Yellow-crowned Euphonia	*Euphonia luteicapilla*	R	O	N	0	1
Yellow-faced Grassquit	*Tiaris olivaceus*	R	O	Y	1	26
Yellow-green vireo	*Vireo flavoviridis*	M	O	N	0	13
Yellowish Flycatcher	*Empidonax flavescens*	R	O	Y	1	1
Yellow-margined Flycatcher	*Tolmomyias assimilis*	R	O	Y	0	1
White-naped Brush-Finch	*Atlapetes albinucha*	R	O	Y	8	12
Yellow-throated Euphonia	*Euphonia hirundinacea*	R	O	Y	12	66
Yellow-throated Vireo	*Vireo flavifrons*	M	I	N	0	2

Following Stiles and Skutch (1989) migratory status is listed as Neotropical-nearctic migrant (M) or year-round neotropical resident (R); dietary guilds are listed as frugivorous (F), omnivorous (O), or insectivorous (I); and foraging strata preferences are listed as ground/understory (Y) or middle/upper canopy (N).

### Modeling approach

Seasonal composition and interseasonal dynamics of avian communities in shade-coffee and in secondary forest were examined using a Bayesian implementation of a multi-species, multi-scale occupancy model [29] and a multi-species dynamic occupancy model [30], [31], (referred to as “multi-scale” and “dynamic” models from here forward). These occupancy modeling frameworks provided estimates of parameters allowing us to compare avian community composition and dynamics between habitat types. The multi-scale model framework provided estimates of (1) occupancy, defined as the probability that a patch is occupied by any species in a particular functional group at least once during the annual cycle; and (2) seasonal use, defined as the probability that any species of a particular functional group occurs in a patch during a season given that it occupies that patch at least once during the annual cycle. The dynamic model by contrast provided estimates of (1) initial use, defined as the probability of a species occupying a given patch during an initial season unconditional on occupancy throughout the annual cycle; (2) interseasonal patch extinction, defined as the probability that a species using a given patch does not use that patch the following season; and (3) interseasonal patch colonization, defined as the probability that a species uses a patch that it did not use in the previous season. In the dynamic model, interseasonal transitions (i.e. patch extinction and colonization, henceforth ‘turnover’) were estimated between each successive season in the annual cycle: 1–2, 2–3, 3–4, 4-1. This required an altered parameterization from the original dynamic model described by [30]. Specifically, each of the four transitions was included in the model, rather than just the first three, which would be standard for a chronological sequence of time periods (e.g. years). As such, this dynamic model becomes a cyclical dynamic occupancy model.

Both multi-scale and dynamic models account for biases induced by variation in seasonal capture probability to evaluate differences in avian community composition and dynamics. Seasonal capture probability, pooled across individual sampling days within a season, was estimated instead of daily capture probability because daily capture rates were too low for estimating daily capture probabilities and occupancy metrics. The submodel for seasonal capture probability for both the dynamic and multi-scale occupancy models included the following factors: (1) species by season interaction, (2) habitat type, (3) diet guild, (4) migratory status, (5) foraging strata preference, and (6) habitat-type by diet-guild interaction. The submodel for occupancy within the multi-scale occupancy model included the following factors: (1) species, (2) habitat type, (3) diet guild, and (4) habitat-type by diet-guild interaction. The submodel for species turnover in the dynamic occupancy model and the submodel for seasonal use in the multi-scale occupancy model included: (1) species, (2) season, (3) habitat type, (4) diet guild, and (5) habitat-type by diet-guild interaction. In each of these submodels, predictors were specified as fixed effects except for the species effect and species-by-season effect interaction. These random effects were specified such that parameters for species or species-season combinations with sparse captures were informed by species or species-season combinations with more frequent captures [31].

We used a Bayesian approach to fit the models using programs R and WinBUGS [32], [33], whereby we specified vague logit-scale priors using a normal distribution with a mean of 0 and a precision of 0.4 for the fixed effects and means for the hyperdistributions on the random effects. For the standard deviation of the random effect, we specified a vague uniform prior with a minimum of 0.001 and a maximum of 10. We confirmed convergence of three Markov-chain Monte Carlo (MCMC) chains each of length 15,000 by visually inspecting the plotted chains and ensuring that Gelman-Rubin diagnostic values were below 1.5 for all estimates [34]. Predicted estimates from these models enabled us to compare avian community composition and dynamics between shade-coffee and secondary forest. In particular, inferences were based on 95% Bayesian credibility intervals (BCIs; 2.5^th^ and 97.5^th^ percentiles from MCMC output) surrounding probability estimates. If a BCI excluded the mean of a contrasting estimate, then this was considered a statistically significant difference. When comparing a higher probability to a lower probability, the following standard formula was used: (higher-lower)/higher.

## Results

### Avian captures

Including recaptures, a total of 1,561 captures representing 73 bird species and 13 guilds were made. Of those, 773 captures representing 61 species were in shade coffee and 788 captures representing 46 species were in secondary forest (Table 2). Of the total birds captured, 217 captures were birds that had been previously captured and banded. This included 115 birds that were re-captured once, 31 birds that were re-captured twice, 6 birds that were re-captured three times, 4 birds that were re-captured 4 times and 1 bird that was re-captured 6 times. The total number of individuals banded during our study was 1,344 and 1,192 of these were only captured once. Fifty-three percent of all bird species captured were captured in only one of the two habitat types: 36% were captured only in shade coffee (27 bird species) and 16% were captured only in secondary forest (12 bird species). Sixty percent of the highly forest dependent species captured were forest insectivores (e.g. Dendrocolaptidae: *Sittasomus griseicapillus*, *Glyphorhynchus spirurus*, and *Dendrocolaptes certhia*; Thamnophilidae: *Thamnophilus doliatus*; Tyrannidae: *Rhynchocyclus brevirostris*, and *Tolmomyias assimilis*). We captured one frugivorous species (*Chiroxiphia linearis*) during the study, which was captured in both shade-coffee and secondary forest. Compared to secondary forest, in shade-coffee we captured a higher number of omnivorous (43 in shade-coffee vs. 30 in secondary forest) and insectivorous species (17 in shade-coffee vs. 15 in secondary forest).

### Occupancy and seasonal use

**Figure 2:**
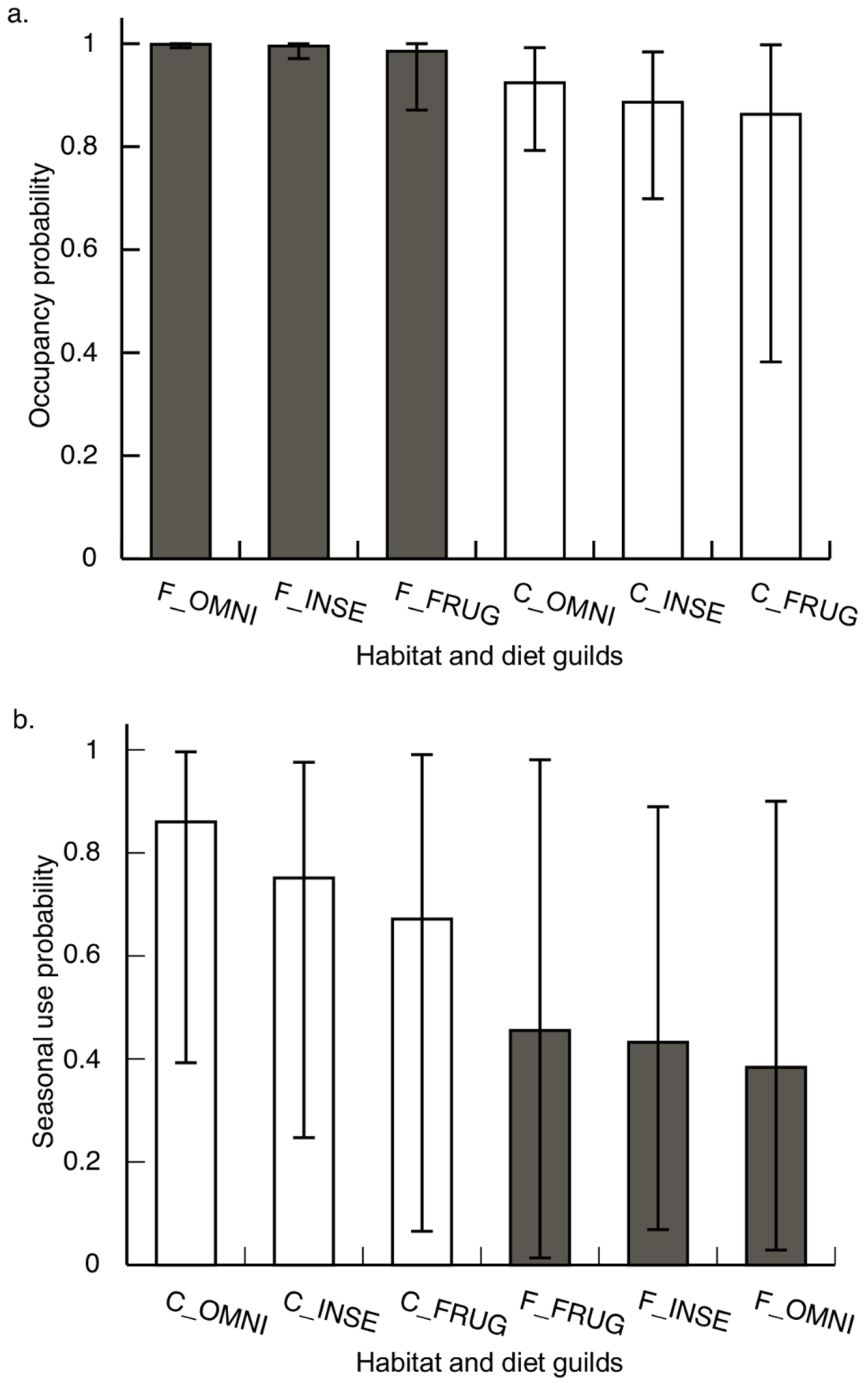
Variation in patch occupancy (A) and seasonal patch use (B) among habitats and avian dietary guilds based on a multi-scale occupancy model fit to mist-net data from four shade coffee plantations and three secondary forest fragments near San Luis, Costa Rica, 2005–2008. Habitat types included forest (F) and shade coffee (C); avian dietary guilds included frugivores (FRUG), insectivores (INSE), and omnivores (OMNI). Whiskers represent 95% Bayesian credibility intervals.

Occupancy estimates were quite high across habitats and avian functional groups (>0.85; Fig. 2a). Omnivores and insectivores were more common in secondary forest than they were in shade-coffee, and these differences were statistically significant. Mean occupancy of frugivores was greater in secondary forest than in shade coffee, but this difference was not significant. Seasonal use was moderate across most of the diet-habitat type combinations (range: 0.38–0.75), except for seasonal use by omnivores of shade-coffee, which was quite high (0.86; BCI: 0.39–1.00; Fig. 2b). Although means for seasonal use were greater in shade coffee than they were in secondary forest, none of the differences were statistically significant because of wide BCIs.

### Community turnover

Omnivores tended to have higher mean interseasonal colonization rates in shade-coffee (0.38; BCI: 0.04–0.88) compared to forest (0.09; BCI: 0.00–0.42), but this difference was not statistically significant. Other functional groups had moderate colonization rates across habitats (range: 0.21–0.59), and comparisons of these rates between habitats were not statistically significant. Estimates for interseasonal persistence were moderate (range: 0.57–0.73), and differences among habitat types and functional groups were not significant.

### Capture probability

**Figure 3:**
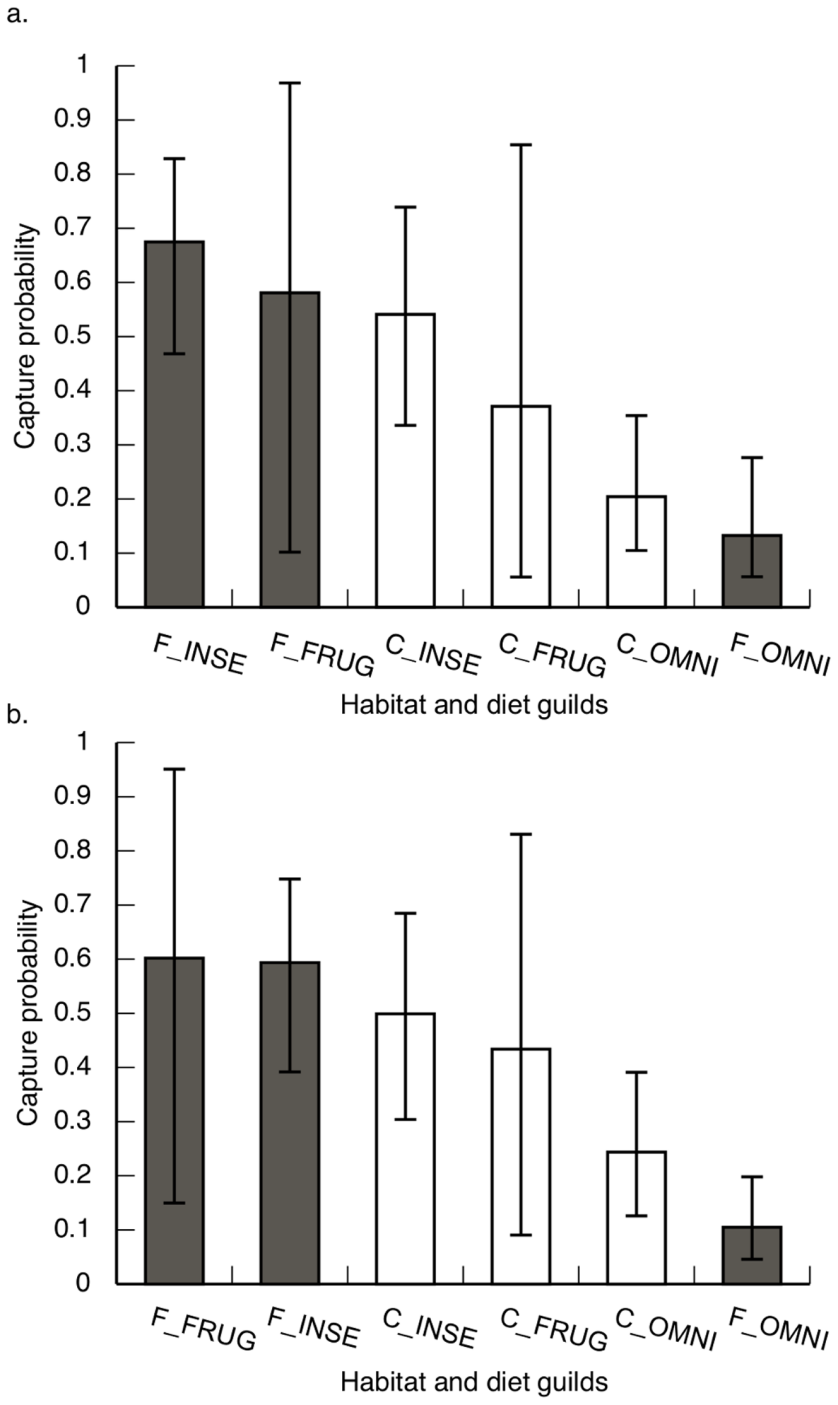
Variation in avian capture probabilities among habitats and avian dietary guilds based on a multi-scale occupancy model (A) and a dynamic occupancy model (B) fit to mist-net data from four shade coffee plantations (white bars) and three secondary forest fragments (gray bars) near San Luis, Costa Rica, 2005–2008. Habitat types included forest (F) and shade coffee (C); avian dietary guilds included frugivores (FRUG), insectivores (INSE), and omnivores (OMNI). Whiskers represent 95% Bayesian credibility intervals.

Capture probabilities were quite variable between shade-coffee and secondary forest, and among functional groups based on the multi-scale occupancy model (range: 0.13–0.67; Fig. 3a). As expected, capture probability estimates based on the dynamic occupancy model were similar to the multi-scale occupancy model (Fig. 3b). For frugivores and insectivores, estimates for capture probabilities were moderate (range: 0.43–0.60) and were not significantly different between the two habitat types. Frugivores and insectivores tended to have higher detectability in secondary forest compared to shade-coffee, but the differences were not statistically significant. For omnivores, estimates for capture probabilities showed a lower mean detectability of omnivores compared to insectivores and frugivores, for both habitat types.

## Discussion

Our study and analyses of bird community composition and dynamics did not refute the hypothesis that coffee agroforestry systems serve as surrogate habitats for forest bird species. The modeling approach we used allowed us to consider the important factors of seasonal use and detectability differences while comparing the ecosystem services provided by birds in shade-coffee to those by birds in secondary forest. Specifically, the analyses revealed that bird community composition and dynamics in shade-grown coffee plantations that are small and embedded in the typical tropical countryside landscape do, in fact, reflect those of secondary forest, and these findings are consistent with the habitat-surrogate hypothesis. With few exceptions, patch occupancy, seasonal use, species turnover, and even capture probability were mostly similar between habitat types.

### Species composition and turnover

Based on raw captures, our study indicated that species richness was higher in shade-coffee compared to secondary forest across the avian community and within the omnivore and insectivore guilds. When we accounted for differences in capture probability, however, species occupancy was higher for omnivores and insectivores in secondary forest compared to shade-coffee. The probability of occupancy for individual species by guild and habitat type is a function of the number of species within a guild occupying a given habitat type and their distribution across patches (i.e., study sites) of that habitat type. As the number of species in patches of a given habitat type increases, the estimated probability of occupancy within that habitat type increases. Probability of occupancy in this context can therefore be considered a proxy for species richness both within and across patches of a particular habitat type.

Although the difference in occupancy between habitat types was statistically significant, occupancy rates in both habitat types were all very high (>0.85) and differences were less than 15%, which we interpret as not a biologically meaningful difference. Thus, we conclude that the high occupancy rates across all dietary-guild and habitat type combinations lends support for the habitat-surrogate hypothesis that bird communities in coffee agroforests represent those in secondary forest. Our examination of species turnover and seasonal use demonstrated that avian communities were quite dynamic between seasons, especially in shade-coffee when compared to secondary forest, but we found no statistically significant differences in these dynamics between habitats. Omnivores, however, displayed evidence that led us to hypothesize a greater likelihood they would colonize a shade-coffee plantation than they would colonize a secondary forest fragment. Despite the one potential exception with respect to omnivore colonization, species turnover patterns were consistent with the habitat-surrogate hypothesis.

### Capture probability

Based on our analysis of bird community composition and dynamics, bird species within functional guilds have similar levels of detectability in shade-coffee and in secondary forest when using mist-nets as a sampling method. Although one recent study has incorporated detection probability to compare migratory bird species densities between shade-coffee and forested habitats [35], our study is the first to account for detection probability when assessing how well these habitat types support the entire bird community [10]. Additionally, omnivores displayed some differences in detectability compared to the other functional groups irrespective of habitat types (i.e. omnivores had a lower detectability in both shade-coffee and secondary forest compared to frugivores and insectivores). Determining which traits of omnivores predispose them to be less likely to be detected in these two habitat types may further demonstrate the need for a standard methodology and analyses for studies of bird community changes in human-dominated landscapes. If we had ignored capture probability, we would have found that species richness, especially within the insectivore guild, was greater in shade-coffee than in secondary forest. When accounting for capture probability, however, we found the opposite to be the case albeit the difference was probably too small to be biologically significant. Nonetheless, our finding that omnivores had lower detectability compared to frugivores and insectivores underscores the importance of considering detection probability when evaluating the contribution of a particular habitat type toward conservation of multiple functional groups.

### Conservation and management implications

In order to make sound conservation and management recommendations for shade-coffee plantations, we must first be confident about their role in supporting forest bird communities. This requires conducting more detailed studies that not only measure apparent species richness, but also aim to evaluate relative abundance, community dynamics, and community composition. Although we did not measure landscape-scale variables, we did conduct our study in a landscape that represents the typical landscape where shade-coffee farms are located, as most coffee agroforestry production systems are smallholder farms. Based on our findings, therefore, shade-coffee still appears to provide a suitable surrogate for secondary forest across the annual cycle, with two exceptions. First, we found that bird occupancy was slightly higher in secondary forest compared to shade-coffee, and this difference was statistically significant for omnivores and insectivores. However, some caution should be taken in extrapolating our results to coffee agroforests that are either very extensive or are within landscapes that do not include forest patches.

Nature tourism has increased dramatically in the last ten years in the Monteverde region of Costa Rica [36] and several economically-important bird species are found in the Monteverde region (e.g. Resplendent Quetzal, Three-wattled Bellbird, and Black-faced Solitaire). Although this region originally gained worldwide acclaim for its three private reserves, most of the region, outside of the reserves, is more typical of tropical agricultural landscapes [21], [37]. Within these areas outside of the reserves in Monteverde, the economic condition of many families is directly related to income from bird-related nature tourism. Therefore, bird research aimed at understanding how well various land use types in the agricultural matrix support bird communities would not only help to guide conservation efforts in the region, but would also be economically beneficial to local human communities. Much research has already been conducted on birds in the Monteverde region, however several factors limit the broad applicability of this research to conservation/management of bird communities throughout the entire region. First, most previous research in the Monteverde region has focused on single bird species [38], [39], limiting application of management recommendations to entire avian communities. Second, study sites have primarily been within protected areas, not in agricultural land use types. Lastly, avian community composition changes rapidly with elevation in the region [22], [40]. For example, many species of the avian community of the San Luis valley, where our study was conducted, do not range into the higher elevations of the Monteverde region [37], [38], [41], [42]. Thus, our study provides important conservation information for the region, demonstrating that agroforestry systems can protect forest bird communities throughout the annual cycle.

Although we did not include a more intensified agricultural system in our study, agroforestry systems can protect more native biodiversity than more simplified agricultural systems [10]. For example, cacao and banana agroforestry systems contain bird assemblages that may be as abundant, species-rich and diverse as secondary forests [9]. Thus, conserving and managing shade-coffee and forest remnants for bird species should be considered as a complementary rather than a contradictory strategy for bird conservation. Management efforts should therefore focus on conserving all forested habitats, including agroforestry systems, to maintain bird species composition and dynamics.
